# Disaster risk, disaster resilience and traditional cultures as collective memory in Central Sulawesi, Indonesia

**DOI:** 10.4102/jamba.v18i1.2094

**Published:** 2026-09-01

**Authors:** Andi Akifah, Andi A. Unde, Syamsuddin Azis

**Affiliations:** 1 Department of Communication, Faculty of Social and Political Sciences, Hasanuddin University, Makassar, Indonesia; 2 Department of Communication, Faculty of Social and Political Sciences, Tadulako University, Palu, Indonesia

**Keywords:** collective memory, disaster risk, Indonesia, Central Sulawesi, Kaili, Kayori, traditional cultures

## Abstract

This article addresses the issue of disaster resilience and mitigation in Central Sulawesi, Indonesia. The province is frequently hit by earthquakes, tsunamis, floods and liquefactions due to its location along the Palu-Koro fault line within the Asia-Pacific Ring of Fire region. Amidst these challenging geographic conditions, the predecessors of local communities in Central Sulawesi have developed a unique system of collective memory to deal with disasters through centuries of life experience. This article examines how local communities in Central Sulawesi preserve and transmit disaster-related memories across generations as disaster resilience and mitigation strategies. It mainly aims to investigate the community’s collective memory regarding disaster mitigation through the traditional cultures of the local communities in Indonesia. A particular focus has been on the province of Central Sulawesi. The research findings indicate that collective memory in the province should have played a crucial role in community preparedness and adaptation, particularly for contemporary disaster risk and disaster resilience strategies. The local toponymy can warn people to avoid geographically and environmentally dangerous areas. Crucially, traditional stilt houses, adapted from past disasters, are believed to be able to survive disasters such as floods, earthquakes and tsunamis. Moreover, oral narratives such as folklore and Kayori (poetry) may serve as an early warning system for the dangers of engaging in activities that go against nature.

**Contribution:**

The collected memories, unfortunately, have not been well acknowledged and regenerated. Evidence from the field research revealed that many of the disaster-related traditional cultures have been forgotten and neglected. This phenomenon is caused by modernisation, urbanisation and degradation of local language skills.

## Introduction

Natural disasters not only leave physical traces in the form of damaged infrastructure but also leave a lasting impression on the collective memories of the community. These memories can sometimes become assets for disaster resilience but often become memories that can cause deep trauma for certain individuals. Traditional cultures, in this study, referring to traditional beliefs, local wisdom, values and social structures, can be a proven effective coping strategy in reducing disaster risk (Alhadi et al. 2023; Firdaus et al. 2023). These traditional cultures can help identify patterns of hazard occurrence and, if shared appropriately within a community, can improve community risk perception (Solekhah et al. 2025). For example, heritage and local knowledge, which constitute the collective memories of a community, also contribute to enhancing community resilience by increasing a sense of solidarity and identity among community members (Fontanella Pisa 2024). Therefore, collective memories may influence the effectiveness of disaster risk management plans (Rozi, Ritonga & Januar 2021). The concept of collective memory has attracted the attention of scholars. This concept has been explored extensively in the context of disaster studies (Kempe 2006; Sutton et al. 2020; Yamada 2022).

Collective memory functions as a social construct that influences how communities interpret and respond to crises (Halbwachs 1950, 1980, 1992). Therefore, collective memory, formed through inherited experiences interacting with nature, serves as an important foundation for building disaster resilience based on local wisdom (Sururoh & Trinirmalaningrum 2018). The idea of collective memory was first introduced by Maurice Halbwachs and has been developed by several other scholars. Collective memory is the process of sharing memories, knowledge and information across a social group that is significantly linked to group identity (Halbwachs 1992; Jara-Figueroa 2016; Tenenboim-Weinblatt & Baden 2016; Yasseri, Gildersleve & David 2022). According to Maurice Halbwachs (1980), collective memory can be constructed, shared and inherited by social groups, large or small, such as a nation, a generation or a community. Collective memory highlights how a society preserves and affirms certain values and ideals. Theorising about memory – what is memorialised and what is omitted – as a way of preserving the past offers insights into the discursive constructions that frame memory at personal, social and collective levels (Littlejohn, Foss & Oetzel 2017). Central Sulawesi province is frequently hit by earthquakes, tsunamis, floods and liquefactions due to its location along the Palu-Koro fault line (see Figure 1). The September 2018 natural disaster was one of the extraordinary tragedies that triggered public awareness of the dangers of disasters. Many analysts and geologists predict that similar disasters could occur again in the future. Amidst these challenging geographic conditions, local communities in the area have developed a unique system of knowledge and collective memory for dealing with disasters through centuries of life experience. The collective memory has become a kind of enigma – a puzzle that continues to be reconstructed from generation to generation.

**FIGURE 1:**
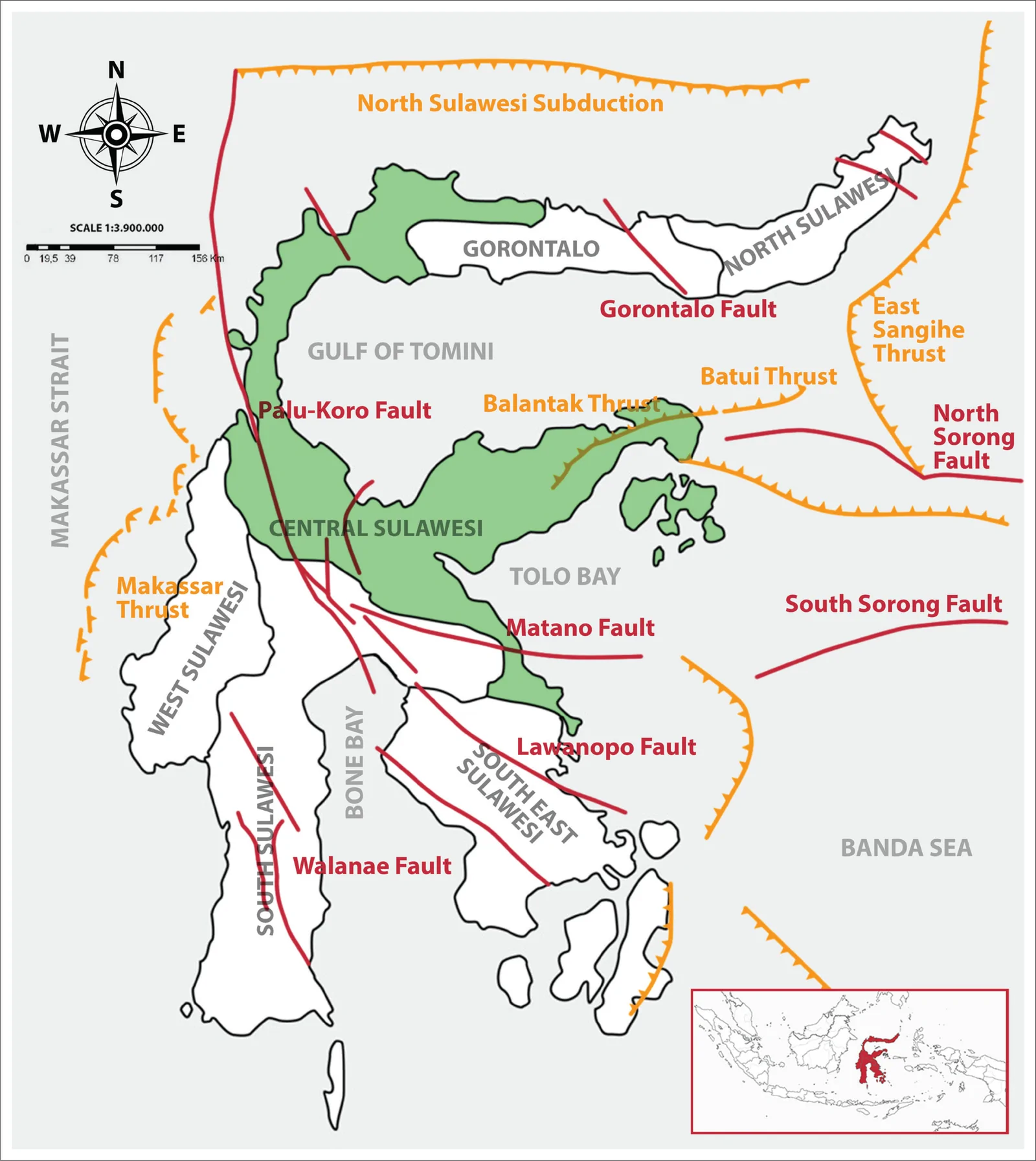
Map of Central Sulawesi with fault sources.

Long before the 2018 disaster, people of Central Sulawesi had their own ways of remembering and passing on memories of the disaster. Local knowledge about this disaster is manifested in various forms, including traditions, spatial practices and building constructions. This study mainly questions how the people of Central Sulawesi remember, interpret and pass on their disaster experiences. This article constructs several important lessons, which we believe constitute the contribution of this study to the body of knowledge, scholarship and wider society. Firstly, it focuses on toponymy. Secondly, it highlights oral traditions. Thirdly, it is about traditional rituals. Fourthly, it examines how traditional houses are constructed as part of disaster mitigation in the area.

## Research methods and design

This research employed qualitative methods by using the phenomenological and hermeneutic approach.

Phenomenology is an approach to describing things as we experience them, long before we formulate them in our minds (Aspers & Corte 2019; Creswell 2013; Gora 2014; Wang et al. 2014). The main characteristic of the phenomenological tradition is the study of the lifeworld as we experience it directly, pre-reflectively, rather than as we conceptualise, theorise, categorise or reflect on it (Given 2008). The phenomenological tradition assumes that people actively interpret their experiences and try to understand the world through their personal experiences (Gora 2014). Phenomenology is concerned with the systematic reflection and analysis of phenomena related to conscious experience, such as human judgements, perceptions and actions, with the aim of: (1) appreciating and describing social reality from the diverse subjective perspectives of the participants involved and (2) understanding the symbolic meanings (‘deep structures’) underlying these subjective experiences (Bhattacherjee 2012). The study also uses hermeneutics (Arthos 2009; Coelho 2002; Creswell & Poth 2018; Grossberg & Christians 1978; Hawes 1977) to assist in interpreting texts in the form of documents, audio recordings and audio-visuals of folklore related to disasters that occurred in the past.

Through interpretive efforts to understand the true reality, researchers can understand more deeply and not be limited to what is presented in the text (Putra 2012). Hermeneutics is used to understand the layers of meaning in the collective memory of the disaster in Central Sulawesi. In this study, hermeneutics is relevant because it examines the interpretative process. Hermeneutics seeks to explain the important concept of interpretation as a continuous and ubiquitous aspect of human experience, defines its boundaries and evaluates its importance.

Therefore, interpretation is a fundamental component of human communication, particularly in hermeneutical activities (Grossberg & Christians 1978; Hawes 1977). This method enables researchers to not only describe what is remembered but also explore how these meanings are constructed, negotiated and inherited in specific socio-cultural contexts.

This study addresses the province of Central Sulawesi, which is situated on the island of Sulawesi, Indonesia (Figure 2). Since the province consists of districts whose population mainly relies on spoken language, the researchers collected data in the form of text, images and videos, as well as building structures. Therefore, our research used interviews to purposively select informants. The criteria set were community leaders or people who have knowledge of local disaster traditions and have direct experience of natural disasters that have occurred in Central Sulawesi. This study collected a lot of data from the Kaili Tribe, considering that this tribe is the largest tribe in Central Sulawesi among other ethnic groups. The locally so-called ***To Kaili*** [Kaili people] predominantly occupy Donggala Regency and other sub-districts in other regencies across Central Sulawesi Province. Based on the population census, the Kaili people, who are widely spread in Central Sulawesi Province, are estimated to make up 45% of the total population of Central Sulawesi (BPS Sul-Teng 2019, 2022, 2023, 2024, 2025; Mattulada 1983; Yetti 2016).

**FIGURE 2:**
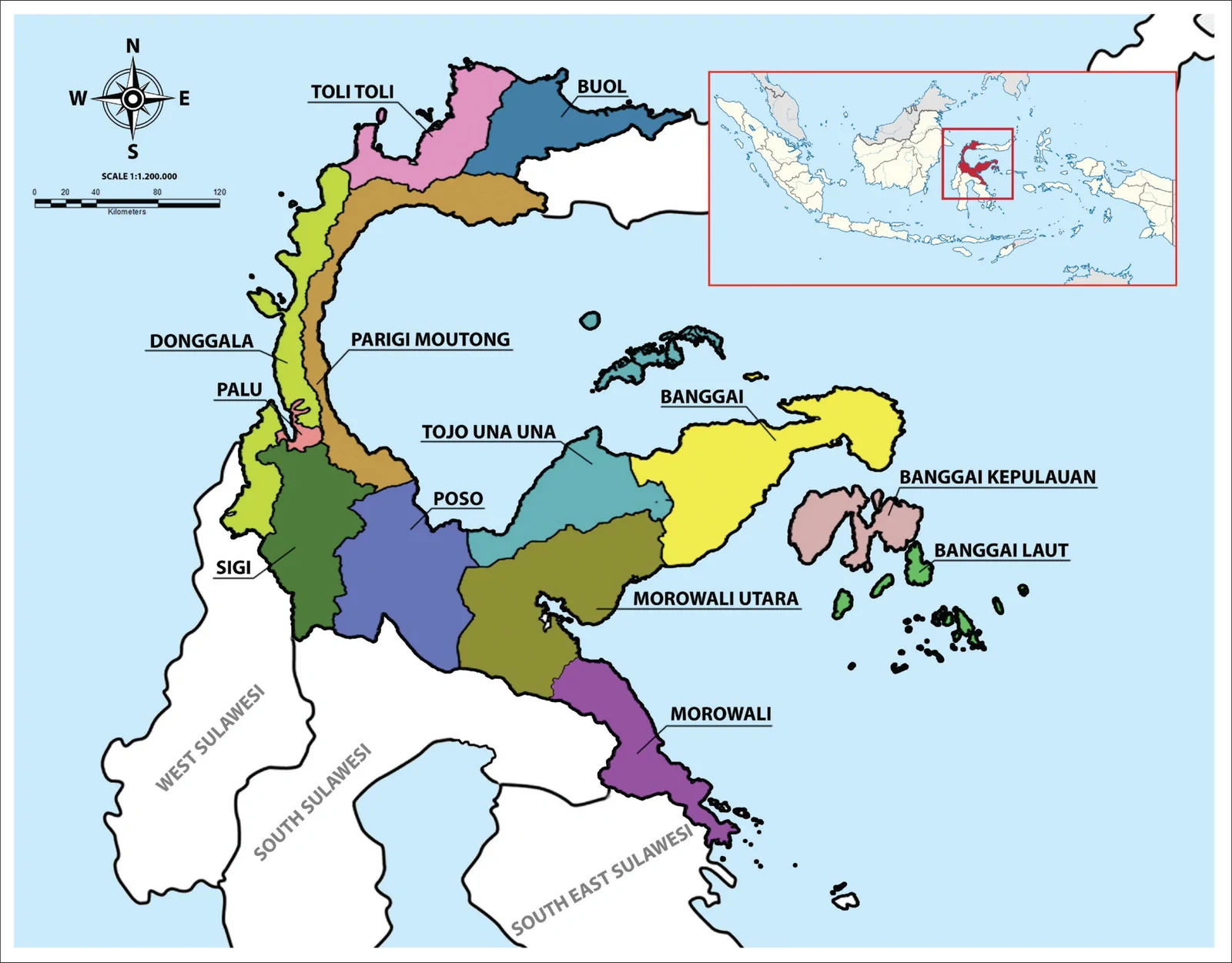
Map of Central Sulawesi, Indonesia.

### Ethical considerations

Ethical clearance to conduct this study was obtained from the Faculty of Social and Political Sciences, Tadulako University and the Central Sulawesi Provincial Government, the National Unity and Regional Political Agency (Ref. No. 130/UN28.1.31/KP.10.00/2026 and 070/0640/BID/III-BKPD/2025).

## Results and discussion

Communities affected by natural disasters, such as those in Central Sulawesi and its surrounding areas, require high vigilance and strong disaster resilience to face disaster risk. Disaster risk is defined as the likelihood that a system, society or community may experience fatalities, injuries or destroyed and damaged assets within a given time frame. This risk is calculated probabilistically as a function of hazard, exposure, vulnerability and capacity (UNDRR 2015). Various communities have emerged to assist affected communities through disaster resilience programmes. Several studies define disaster resilience as a key capacity related to a community’s adaptive nature to disasters and post-disaster response and development (Bakić & Ajdukovic 2019, 2021; O’Neill et al. 2016; Sarker et al. 2020). Furthermore, disaster resilience can also be defined as a process that connects a network of adaptive capacities (resources with various attributes) to adapt after a disaster (Norris et al. 2008; Sherrieb, Norris & Galea 2010).

For the Kaili people, the majority ethnic group in Central Sulawesi, disasters are not merely physical events but are embedded in cultural narratives that guide adaptive behaviour (Afiayati 2022; Palogai & Bohang 2021).

By thoroughly observing some documents, videos and literature on traditional cultures, it is found that the community of Central Sulawesi is indeed rich in disaster-related cultural heritage as their collective memory of disaster. These collective memories are manifested in various forms, including oral narratives such as folklore; a local poetry, which is so-called *Kayori*; a regional naming system called toponymy and building construction, such as traditional stilt houses. The field data evidently show several important lessons on how natural disasters are closely related to collective memory in Indonesia and the world.

### Local naming (Toponymy) as a memory archive

One form of collective memory among the people of Central Sulawesi is toponymy or regional naming (Afiayati 2022; Maliki 2022; Suntoro et al. 2023). The toponymy is based on events that have occurred in the area, including disasters. The toponymy crucially indicates that the Kaili people, as the largest ethnic group in Central Sulawesi, understand which areas have historically and topographically experienced disasters and identify disaster-prone areas based on their names (Yetti 2016). Several areas in Palu, Sigi and Donggala have been marked by disaster terms, such as ‘*Kaombona*’, which has become better known as ‘*Tanah Runtuh*’, meaning areas that have previously experienced landslides and collapses. Evidently, the 2018 earthquake shows thousands of houses and buildings in the province were massively damaged.

Nene Baca (85 years old), a traditional elder in Tondo of Palu, said that several areas that are currently densely populated around Tondo and Talise were previously places that people avoided settling because of their history of being known to be prone to disasters (Figure 3). He put it this way:

‘For example, the *Tanaruntu* area around Tondo experienced a landslide in 1953, coinciding with the collapse of the *Tururuka* road on the Palu-Parigi axis. In the fishing village, there used to be a straight road that continued all the way to Tondo, and it collapsed first. ‘Collapse’ is called ‘*naombo*’ or ‘kaombona’ in our language. There was no earthquake at the time, but it suddenly collapsed, they said, because there was a hole there.’ (Interview with Nene Baca in 2025, male, a farmer, 85 years old)

**FIGURE 3:**
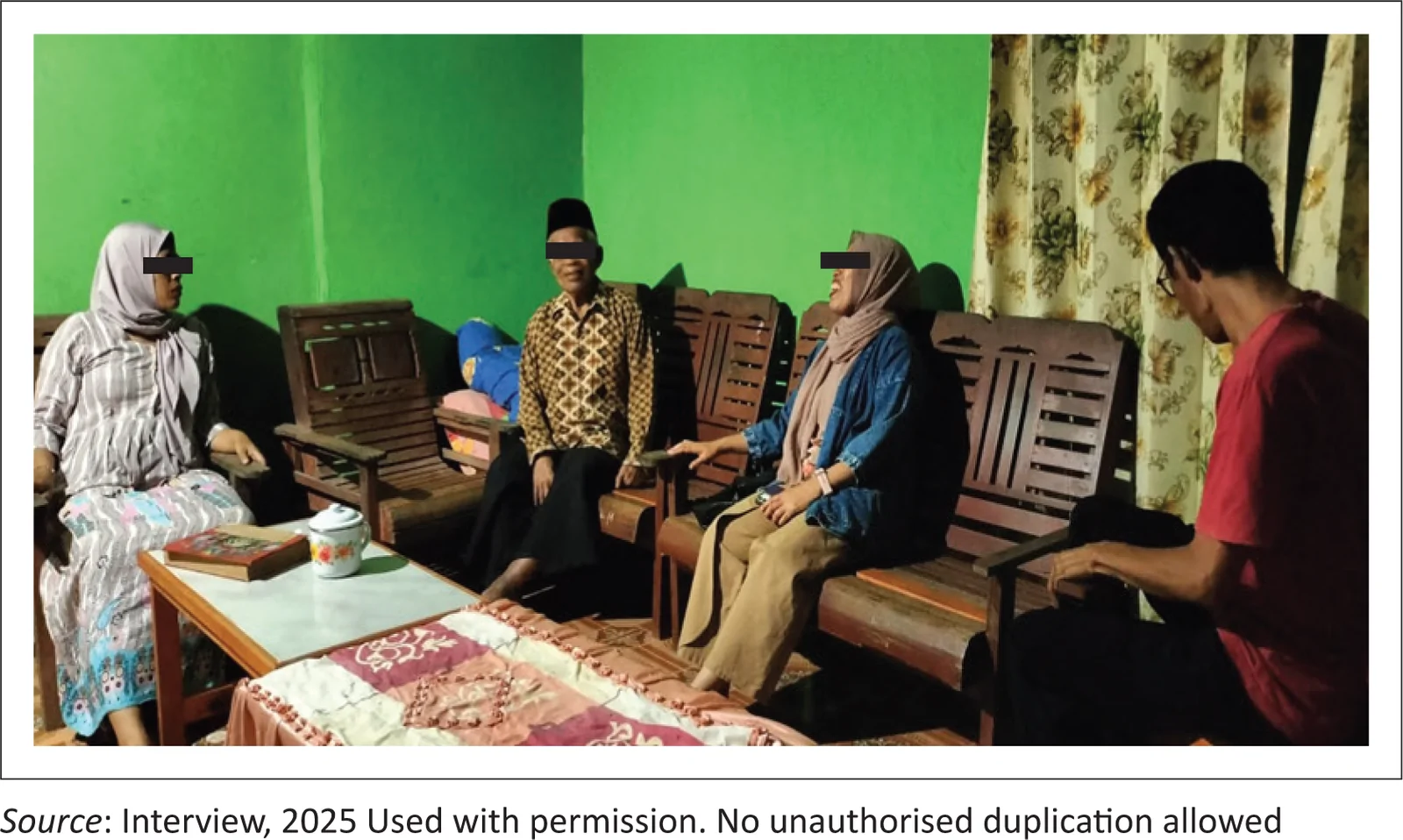
A key informant in Tondo village of Kota Palu.

Empirical evidence from the field indicates that there are 100 names of places that refer to and contain disaster meanings. For example, the name Palu is a toponym.

Originated from the word ‘*Topalu’e*’, which literally means uplifted land. Later on, Palu became the capital city of Central Sulawesi Province. The Kaili believed that the Palu area originated from the ocean, which then formed land from shifting plates due to earthquakes (Palogai & Bohang 2021). Traditionally, the Kaili use toponymy to perpetuate memories of danger, effectively marking vulnerable locations as avoidable zones.

This place-naming practice serves as a social-spatial framework that guides community behaviour in preserving their historical collective memory about the local natural environment. Therefore, the impact of potential damage and losses from disasters can be reduced if local knowledge is understood, acknowledged, recognised, implemented and applied as a reference in development or other disaster risk reduction measures (Sururoh, Trinirmalaningrum & Lubis 2022). In traditional societies with limited mobility, such knowledge is vital. Someone new to a particular area can quickly understand the risk topography simply by hearing the place names – provided they understand the language, memory and cultural context. Halbwachs (1950) explains that physical space is one of the most important frameworks for collective memory. According to Halbwachs, landscape is not neutral – it becomes a ‘memory space’ that helps communities remember their past. Understandably, therefore, when the Kaili frequently name places and areas that contain commemorative symbols, they are not only remembering past events but also reproducing practical knowledge about how to live in and deal with the threat of disasters. Unfortunately, amid the rapid modernisation and urbanisation that have swept Palu since the 1980s, the meaning behind these names has been forgotten. Newcomers and new generations do not understand the Kaili language. Younger generations who grew up speaking Indonesian have lost access to the ‘warning codes’ embedded in the toponymy. The development of massive settlement in Balaroa and Petobo, for example, had been carried out without considering the warnings inherent in the place names themselves.

### Oral traditions: Folktales, legends and Kayori

Oral traditions play a crucial role in preserving the collective memory of disasters. In various communities in Central Sulawesi, stories about powerful earthquakes, large waves sweeping across the coast, landslides and land moving are passed down from generation to generation through narratives that are often wrapped in the form of legends or myths. The process of the sea drying up or receding in Kaili Bay is thought to have occurred long before the 16th century, because in the 16th century there was already the Kingdom of Palu (Mamar, Mappalahere & Wayong 1985). The story about the receding Kaili Bay and places is mentioned in various versions. The research data indicate that there are two main sources of collective memory studies in the region. Initially, it is connected to literature from South Sulawesi Province. It is particularly the Epic of Sureq I Lagaligo. For example, one of the popular stories was about a fight between the dog belonging to Sawerigading (*Saverigadi – in* the Kaili dialect) (the central figure in the Sureq epic Lagaligo, South Sulawesi), named ‘*Labolong*’ [the black one], and a giant eel. The fight occurred when the Sawerigading ship was anchored in Donggala. In the story, while walking on Sigi land, without realising it, Labolong’s foot was trapped in one of the holes where a giant eel (Lindu) lived. There was a fierce fight between the two; because of its enormity, it seemed as if an earthquake had occurred, shaking the earth (M. Arif, Djorimi & Abubakar 2019; M. Arif et al. 2020; Mattulada 1983). La Bolong managed to catch the eel out of its hole. After emptying and collapsing, the large hole where the eels once lived became a lake, which is now called Lake Lindu. Next, Labolong took the eel away while it was thrashing and made a hole in the form of a channel through which strong seawater flowed. The fast-flowing water was like spilled floodwater, causing the Kaili Sea to dry up. As a result, the bay, which was previously a busy shipping area, eventually dried up and became land as seen today as the Palu Valley (Abubakar 2019; Mattulada 1983). The myth is still often told by local elders in the Kaili valley, half of whom consider it to be a true story. The second source is local Kaili people who pass on their memories through oral tradition. A pivotal example is the Kaili local story related to the disaster that was also immortalised in a *Tutura*, namely the story *Legenda Tagari Lonjo* [Legend of Tagari Lonjo]. This legend tells about the *Balaroa* area, which is one of the liquefaction disaster areas, formerly known in the Kaili language as ‘*Tagari Lonjo*’. *Tagari* means area, and *Lonjo Na’* means embedded or swampy area (Damayanti et al. 2021; Dwitriansyah, Bo’do & Herman 2024). In ancient times, the Kaili were sometimes prohibited from passing on the dangerous places; for example, they were afraid of drowning if they crossed the swampy road. As the name suggests, the Lonjo area has an unstable soil structure due to the low density of the soil; therefore, heavy loads on it can lead to its collapse (Sururoh & Trinirmalaningrum 2018). Apart from that, in the eastern part of the *Lonjo* area, it is also known as *Tonggo Magau*, which means the place where the King of Palu’s buffaloes wallow (Siagamin 2020). However, as the city develops and the need for housing increases and new immigrants emerge, the people of Palu City seem to neglect this prohibition. In fact, in 1980, the government, together with investors, began to develop the area by evicting people and filling up land so that the soil structure became hard and unsuitable for use as a settlement.

Nowadays, the area is known as *Perumnas Balaroa*. Furthermore, *Balaroa* is the area most affected by the disaster, namely liquefaction on 28 September 2018. Around 1400 houses were destroyed by the earth due to the liquefaction disaster in Balaroa (Sururoh & Trinirmalaningrum 2018). Meanwhile, the area affected in *Balaroa* is around 47.8 hectares out of a total of 238 hectares (Harianto 2020). A disaster survivor whose house was submerged in mud at Perumnas Balaroa said that the house he had lived in since childhood was the house left by his parents. Since childhood, he had never heard or known that the location of his house in Balaroa was in a disaster-prone area that could not actually be used as a residence:

‘Previously, there was no ‘*kaombo*’ story. But if you say that the local community knew, that’s not true; that’s the story after the disaster happened. There were those who mixed it up with the myth.’ (Helmi Hidayat, on 11 October 2025, male, an entrepreneur, 52 years old)

According to Helmi Hidayat, knowledge about disaster-prone areas is not well disseminated in Central Sulawesi. Stories about local wisdom only started to emerge after the 28 September 2018 disaster occurred. Another local folktale or legend that serves as a reminder of disasters is *kayori*, that is, rhymes or poetry, whose content tells about disasters that have occurred in the past. The poem below tells the story of the disaster in 1938:

*‘Goya-goya gontiro… Toka bonga Loli’o*

*Palu, Tondo, Mamboro…Matoyomo….Kamolue melantomo’*.

Meaning:

Rocking in Ganti Village (Banawa, Donggala)

Looking down, Kabonga Village people and Loli Oge,

Palu, Tondo and Mamboro have sunk

What remains is Kayumalue floating

The Kayori tells the story of the earthquake and tsunami in Central Sulawesi in 1938. At that time, part of Mamboro Village sank to the bottom of the sea. Apparently, the disaster repeated itself on 28 September 2018. It is also mentioned that Kayumalue Village is considered safe from disasters. Kayumalue Village is an area located near Mamboro and Tondo. Kayumalue was one of the areas not affected by the tsunami and liquefaction although the effects of the earthquake were still felt. Notably, the *kayori*’s rhymes about the disaster are an extraordinarily important cultural artefact. In Heidegger’s hermeneutical perspective, the ‘living’ text is not just a historical documentation of past events but rather an ‘opening’ [*Erschlossenheit*] of the way of being [existence] of the Kaili people in experiencing, understanding and interpreting the disaster. This text is a manifestation of *Dasein* [human existence] dealing with mortality, destruction and existential uncertainty.

The following interpretation is based on Heidegger’s hermeneutical model.

First line: ‘*Goya-goya Gantiro*’ [Earthquake in Ganti village]. The rhyme ‘*goya-goya*’ is a phrase that imitates the sensation or sound of an earthquake – vibration, shaking, instability. This is not just an external description but an articulation of embodied experience – the body feeling the ground shaking, the world losing its stability.

In Heidegger’s hermeneutics terminology, this is an understanding that is not cognitive-abstract but practical and existential. Kaili people ‘understand’ earthquakes not through seismological explanations but through the experience of bodies being shaken and losing balance. This understanding is pre-ontological – existing before conceptualisation or theorisation. ‘*Gantiro*’ [in Ganti village] provides a specific geographic location.

This is important in Heidegger’s hermeneutic model because place is not a geometric abstraction but an existential space full of meaning – ‘the place where we are’. The earthquake that occurred ‘in *Ganti*’ was not just a geological event at certain coordinates but an event that shook the world. According to Heidegger, human nature is ‘to exist in a world’ [*in-der-Welt-sein*]. The word ‘in’ in these words cannot be given the meaning of simply occupying a certain space at a certain time (Gora 2014).

Second line: ‘*To Kabonga Lolio*’ [They are also felt in *Kabonga* and *Loli*]. This line reveals the extent of the impact – the earthquake was not only local but regional, felt as far away as *Kabonga* and *Loli* (other areas in Central Sulawesi). In Heidegger’s hermeneutics, this can be read as the articulation of a collective affective disposition or collective ‘mood’.

In this context, earthquakes or disasters create a collective mood – the entire region is ‘tuned’ to the same frequency, experiencing the same shaking. Therefore, disasters create a communal awareness that ‘we are all in this disaster together’. Suffering and fear are not individual but communal.

What is interesting about the Kayori rhymes is that it uses the term ‘to’ (them) – not ‘we’. This may indicate a narrator’s perspective outside the three locations mentioned, or it may create narrative distance that allows for the objectification of the traumatic experience. In the context of trauma, narrative distancing is often a mechanism for coping with earthquakes or disasters.

Third line: ‘*Palu, Sigi, Mamboro, Notayomo*’. In this context, the third line is the narrative climax – three locations are mentioned by name, followed by the words ‘*notayomo*’ [‘it has sunk’].

Naming in Heidegger’s hermeneutics is a fundamental form of *rede* [discourse]. By naming places – *Palu, Tondo, Mamboro* – the rhymes significantly and apparently do more than just geographical identification. The rhymes simply and directly mention the communal identity, collective memory and existential significance of these places.

Furthermore, ‘*Notayomo*’, which simply meant ‘already drowned’, is a word that carries a terrible finality.

In these rhymes, ‘drowning’ is not just a physical description (submerged in water), but it is a metaphor for total annihilation, that is, the loss of the familiar world. In Heidegger’s language, this is a world that is collapsing; a world that previously provided context and meaning for existence is now collapsing or liquefying.

Fourth line: ‘*Kayumalue melantomo*.’ If ‘notayomo’ [already sinking] is a confrontation with nothingness, ‘*melantomo*’ [floating] is an affirmation of existence amidst the threat of destruction. In this context, ‘floating’ can be considered a survival mode. The rhyme is a powerful philosophical metaphor.

Floating means surviving with uncertainty (staying above water but not stable). This is a mode of existence that Heidegger might call ‘authentic’: full awareness of vulnerability yet still projecting the possibility of survival.

The specific mention of ‘*Kayumalue*’ is particularly significant. In the Kayori structure, the first three places ‘sink’ (Palu, Tondo and Mamboro), while one place specifically meant ‘floats’ [*Kayumalue*]. Thus, it creates a dramatic contrasting structure: (1) Kayumalue becomes a place of hope – in the midst of total destruction, there is one place that survives. (2) Witness – the survivors become witnesses to the lost. (3) Memory bearers – those who float are those who will narrate events.

From the perspective of Heidegger’s hermeneutics, the place name ‘Kayumalue’ is not an arbitrary label. Names carry the history of the community that lived there, the relationships that existed and the events that occurred. When Kayori says ‘*Kayumalue melantomo*’, it is not just saying that a group of people survived, but it specifically refers to a world or place with all the complexity of its relationships and meanings – still existing even though it is threatened.

In this regard, Edwan, one of the informants who was born and lives in Kayumalue, expressed his pride in his homeland: ‘Our land is blessed’. Edwan admitted that, although it cannot be said to be completely safe, historically Kayumalue has been an evacuation place several times when disasters occurred. In this regard, we learn that several oral traditions regarding the disaster have proven that the ancestors of the people of Central Sulawesi have provided many instructions about their vulnerability and territory. Therefore, they are always alert and protect nature and the environment. Ultimately, disasters do not and will not cause major destruction.

However, Edwan also admitted that the traditional cultures regarding disasters owned by the Kaili community only started to be widely discussed after the 2018 disaster. Before the disaster, local wisdom was not a concern of the community. According to him, the lack of public interest in traditional cultures, including local languages, is the reason why local wisdom is not popular among local people. Moreover, only spoken culture without the support of written culture is an obstacle in preserving local wisdom. So, if reciters of *Kayori, dadendate* or other types of speech no longer exist, it will be difficult to find local wisdom again. As a Kaili person, Edwan admits that he is pessimistic about preserving Kaili’s traditional cultures. Currently, the information digitalisation system, with the help of the Internet, is quite helpful in preserving traditional cultures. Several *kayori* texts and folklore about disasters have been documented in text and video form several parties via various Internet platforms. Even though the number is still very limited, it can reduce pessimism about the fading of Central Sulawesi’s traditional cultures. There are many other kinds of *kayori* related to disasters. However, due to limited space, several rhymes of the *kayori* cannot be included in this article.

### Traditional rituals as collective memory practices

Traditional rituals such as ceremonies to reject evil or offerings to nature spirits are also forms of living collective memory. These rituals are not only spiritual expressions but also mechanisms to maintain communal awareness of their vulnerability to natural disasters.

Empirical findings from the field show that local wisdom implemented through customary regulations for managing natural resources can increase resilience to disasters. This is proven by the 28 September 2018 disaster experienced by the people of Toro Village, Kulawi sub-district. The people of Toro Village had sufficient food reserves to meet their needs during the emergency response period. Therefore, Toro Village distributed the rice aid allocated to them to neighbouring villages. Independence in facing disasters is illustrated in villages in the Kulawi sub-district, where people learn to adapt to an environment full of disaster threats from the experience of their ancestors. Customary regulations regarding the use of forest areas in the Kulawi region increase awareness of landslides and flash floods as well as guide implementation of disaster mitigation actions (Sururoh & Trinirmalaningrum 2018). The Kaili community, in preserving the environment, treats traditional rules with the term *Ombo*. The definition of *ombo* in the Kaili-Ledo spoken language is something that causes disaster because it violates a taboo or is a disaster. The *ombo* in the regulations reflects the cultural values or local wisdom of the Kaili tribe, which must be adhered to in maintaining harmony with nature and the environment (M. Arif et al. 2020). In this regard, a cultural observer emphasised three main principles of the Kaili’s traditional rituals: maintaining a relationship with the Creator, maintaining relationships with fellow humans and maintaining relationships with nature (Iksam Djorimi, interviewed in 2025). These three things are believed to provide security and safety; therefore, a community can avoid or survive danger when disaster strikes. Halbwachs posits that collective memory requires repetition and ritualisation to endure. He asserts that the past can be comprehended through symbols and rituals, alongside historiography and biography (Halbwachs 1992).

Consistent ritual practices establish a ‘temporal rhythm’ that reinforces communities’ ties to nature and the historical calamities of their locale. Through these rituals, younger generations not only ‘hear’ about past disasters but also ‘experience’ an emotional and spiritual bond with that history. Furthermore, Maurice Halbwachs (1992) points out that rituals are one of the most powerful ‘social frameworks’ [*cadres sociaux*] for maintaining and transmitting collective memory across generations. In the context of disasters, these rituals serve as mnemonic devices that activate communal awareness of geological risks and time-tested adaptation strategies. Various traditional cultures (folktales and legends), rituals and buildings emphasising environmental protection indirectly teach about sustainability. Their adherence to the sacredness of natural resources shapes their attitudes and behaviour, leading them to refrain from violating customary laws.

Violations are believed to result in ‘*katula*’ [a form of punishment] in the form of illness, disability or death.

Therefore, to preserve the potential of natural resources, such as Lake Lindu, forests and certain taboos, those affected are subject to sanctions in the form of fines, *dulam* [a type of cloth], *mbesa* [a type of cloth] and other animals [cows or buffalo]. This is reinforced in the tradition by the mottoes ‘*makono ada, malompe todea*’ and ‘*maroso ada, malino ngapa*’ [‘a type of law that is enforced fairly’]; meaning, if the law is enforced fairly, the people will prosper, and the country will be safe and peaceful [Timbang, Malik & Julia 2024]. Consequently, it indirectly reduces and mitigates the risk of disasters.

### Traditional houses as disaster-resistant buildings

In Central Sulawesi, hidden traditional cultures, practices and buildings are contained in every piece of wood and weaving in the traditional houses of the Kaili and Lore tribes. Two iconic buildings, *Lobo* [big house or traditional house] and *Tambi* [family residence], are not just places to take shelter. These traditional house models are architectural masterpieces designed with a deep understanding of nature, including mitigating disasters that often hit this region (see Figure 4). Empirical findings from the field show that the structures of these two houses although they have slightly different functions and demonstrate the same basic principles: resistance to earthquakes and liquifaction, adaptation to tropical climates and harmonisation with the environment.

**FIGURE 4:**
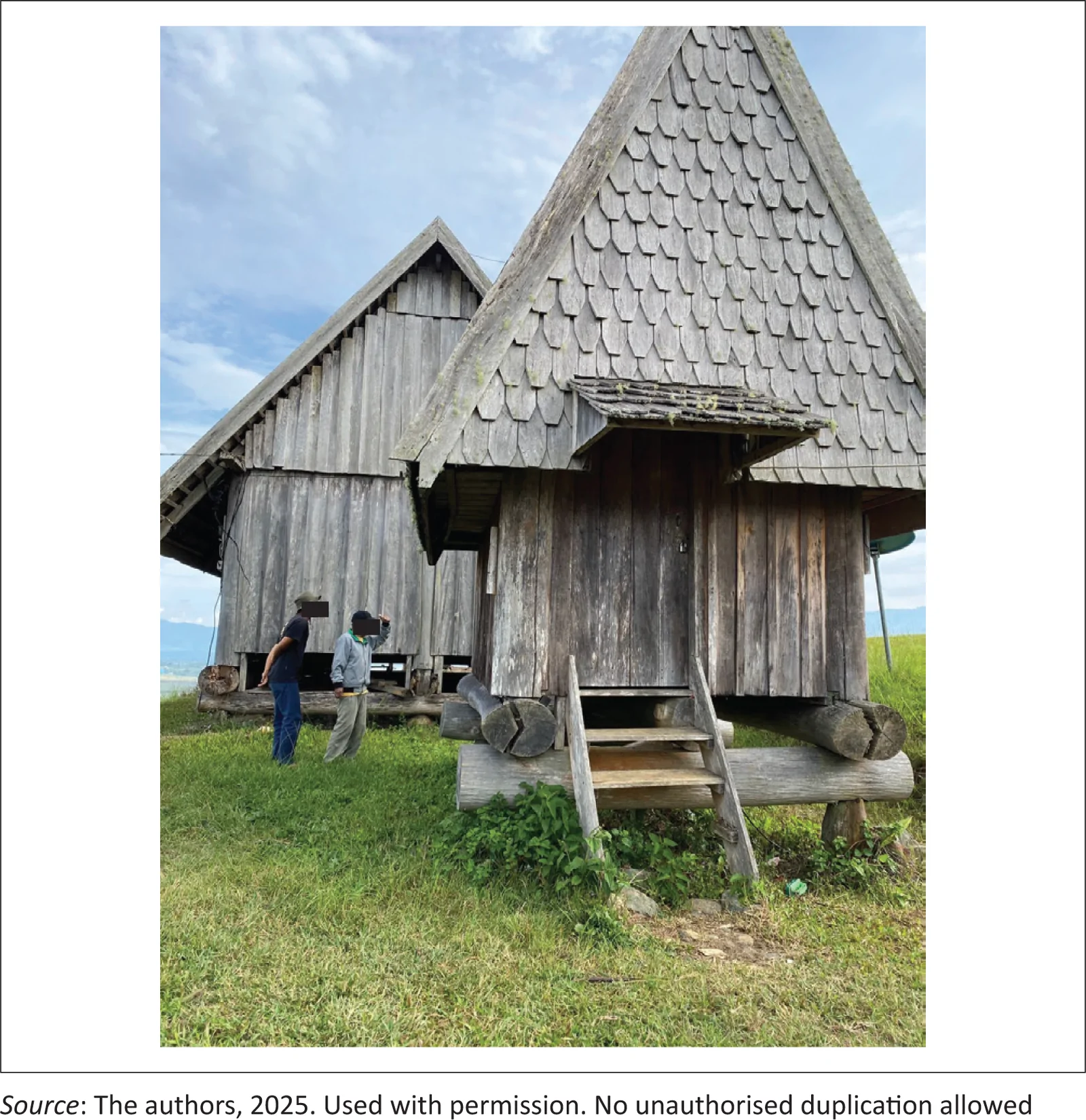
Tambi Traditional House in the Napu Valley, Central Sulawesi.

Figure 4 shows a traditional Tambi house in the Napu Valley. This Tambi house is also used by the Kaili tribe across Donggala Regency. Typically, the Tambi is a stilt house inhabited by a nuclear family.

Its distinctive feature is the soaring roof, which almost covers the entire building, giving the impression of being compact and sturdy. The Tambi is characterised by a saddle-shaped roof with a strong, sturdy wooden frame. This wooden frame is firmly arranged in tiers. The purpose of this design is to strengthen the roof construction. Meanwhile, the walls of the Tambi are made of woven bamboo tied with rattan rope or tree roots.

In the Kulawi area of Central Sulawesi, there is another traditional disaster-resistant type of house similar to Tambi. Local community buildings in the area, at first glance, have more or less the same construction, but if studied carefully, reveal that the differences between houses, including worship and holy places, are quite large; these differences may be due to tribal relations and give an idea of the influence of foreign culture (Schrauwers 2019). Notably, almost all traditional houses of the local people of Central Sulawesi in ancient times had foundations that rested on stones that were half in the ground. The foundation is supported by 2-metre-high pillars driven into the ground. The houses of relatively strong construction were probably built after the great earthquake in 1909, when several villages were more or less damaged and destroyed. This type of house is the most common in the Palu valley. A scholar mentioned that local people have derived the model from their neighbours to the north, but it may have originated in China (Schrauwers 2016, 2019). Apart from residential houses, the people of Central Sulawesi also have traditional building models that are used together. The building called Lobo functions as a traditional hall, meeting place and ceremony.

The structure is larger and more magnificent than Tambi but retains the same architectural philosophy and principles. Walter Kaudern (1925), in his notes, which were the result of his expedition to Sulawesi from 1917 to 1920, considered the Lobo as a temple [*kuil*] because at that time it was often used as a place of worship. Our research notes from the field show that the traditional building construction of the people of Central Sulawesi is believed to be adapted to the geographical and geological conditions of the region. Firstly, the concept of a house on stilts and a fulcrum foundation (joints). This building does not use a rigid stone foundation but is supported by large wooden pillars planted on flat stones as a base. This system functions like a supporting foundation. When an earthquake occurs, the entire structure of the house can ‘sway’ flexibly with the seismic waves, thereby reducing the risk of collapse. The base stone also prevents the wood from directly touching moist soil, thereby preventing rot. Secondly, the joints are made of pegs and ties, not iron nails.

The entire Lobo and Tambi framework is held together using a system of wooden peg joints [*tutu*] tied with rattan or natural fibre rope. This technique creates elastic joints. Therefore, when shaken by an earthquake, these joints have some flexibility and adjust to move, then return to their original position. This is in stark contrast to rigid joints made of iron nails, which can break under massive pressure. Thirdly, a light and firm frame structure. As can be seen in Figure 5, the main material is selected wood that is strong but light, such as ironwood. Roofs made from thatch leaves or palm fibre are also very light. With a structural load that is not too heavy, the inertia force during an earthquake becomes smaller, thereby reducing damage. The various advantages possessed by traditional buildings, especially in terms of disaster mitigation, do not mean that this form of building is still maintained by the people of Central Sulawesi. Currently, traditional house models can only be seen in certain locations that are designated as cultural heritage. However, some concrete buildings have adopted a round foundation model, with intact wood on the lower side of the building. The foundation model has proven its ability to support buildings. Most buildings that used this model did not suffer much damage when the earthquake occurred in 2018, as seen in several buildings in Central Sulawesi. In the *Structures and Settlements in Central Celebes* (Kaudern 1925; Schrauwers 2019), several researchers show that buildings in Central Sulawesi are divided into three categories. Firstly, the sacred hall [*Lobo*], which includes seven types: Kulawi, Pipikoro, Mopahi, Lindu, Towulu, Benda-Besoa and Boku. Secondly, residential buildings, which include three types, namely the Kulawi type, the Napu-Besoa-Koro type and the Kaili-Sigi type. Thirdly, the Lumbung, which includes two types, namely the *gampiri* type and the *paningku* type. Schrauwers mentioned that the differences between the three forms of residential buildings are caused by the structure of the lower part of the house, which influences the shape of the upper building (Schrauwers 2019). The architecture of the Kaili tribe’s shelters is a cultural heritage of the Kaili people, reflecting the local wisdom of this ethnic group. Many shelters are known and introduced to the community, such as *Tambi, Souraja, Paningku, Gampiri, Lobo* and several other names adapted to their respective regions. All types of houses have different functions.

**FIGURE 5:**
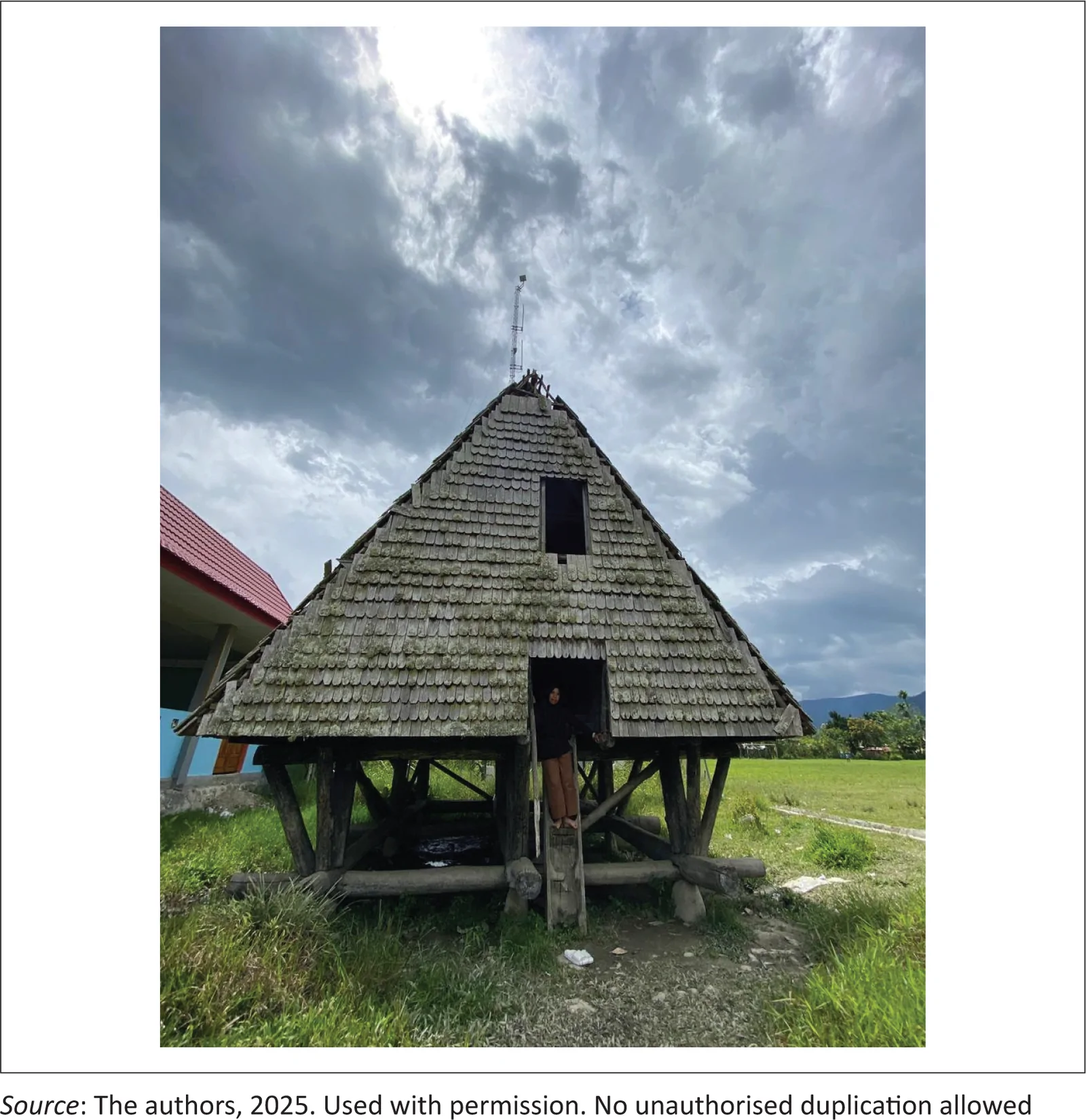
Lobo Traditional House in Sedoa, Poso Regency, Central Sulawesi.

However, the materials and construction are nearly identical. Traditional houses are primarily made of wood. Natural materials such as hardwood and *rumbia* leaves represent memories of familiarity with the local environment (Vellinga 2006). To improve rain resistance, the roofs are covered with a layer of *ijuk*, a type of black fibre, similar to horsehair, found on sago palm leaves (Schrauwers 2019). Although the styles and names vary across regions in Central Sulawesi, the structures of the houses are pretty similar. All of these buildings are constructed with disaster-resistant construction. Kaili architecture is responsive to the local environment and climate, as evidenced by the buildings’ stilt-like structures and sloping roofs, suited to the tropical climate (Zubaidi 2009). The overhanging roofs, which almost touch the ground, crystallise memories of rainfall intensity and strategies for protection against natural elements. The foundation system, with its base stone, captures the collective memory of earthquake experiences, where structural flexibility is prioritised over rigidity. The traditional houses of *Lobo* [shared house] and *Tambi* [residence] in Central Sulawesi are not merely physical structures but are cultural manifestations that record and transmit the collective memory of the Kaili and Lore tribes. The lower part of the Kaili buildings, whether residential, communal, worship or storage, has almost the same philosophy, with the same pillar shape, a natural stone base, and the use of ‘Loanga or Pareva’, namely long and wide beams that depict the very close kinship of the Kaili people.

Furthermore, it has ‘*Nepulanga*’, or beams in a row, depicting the unity of command from the oldest. The ‘*Potanje*’ pegs symbolise the close ties between fellow stratification groups of the Kaili people. Meanwhile, the stairs are made of hardwood sheets (Arifin 2010). Bitterly enough to say that the various advantages of these traditional buildings have not prevented them from being maintained by the community. Many factors have led the people of the province to no longer use traditional house models. Among them is the increasing scarcity of authentic *Lobo* and *Tambi* building skills. Furthermore, the high cost of wood (Susanti et al. 2022), a shortage of skilled workers and the onslaught of modern materials have led to these traditional houses being increasingly replaced by concrete structures, which are not necessarily safer against earthquakes.

However, the tradition contained within them is increasingly relevant. Principles such as flexibility, lightness and harmony with nature should inspire modern development in the province. Integrating modern engineering with local wisdom can produce safer, more comfortable and more sustainable house designs.

Traditional buildings serve as the most robust material framework of the collective memory of the people of Central Sulawesi. This implies, firstly, preserving the memory of disasters: Every architectural detail holds collective memories of past earthquakes, torrential rains and floods. Their form is a ‘petrified memory’ (or ‘wooden’) of how their ancestors survived and adapted. Secondly, transmitting values and knowledge: These buildings and houses are a tool that transmits local wisdom values – such as flexibility, preparedness and harmony with nature – to new generations. By seeing and inhabiting these houses, the collective memory of a resilient way of life is continuously reproduced. Thirdly, the loss of a vital material framework can weaken community resilience because they lose a physical ‘guide’ full of wisdom. Switching to rigid concrete houses without understanding local natural mitigation principles is a sign of a break in this collective memory. The empirical findings from the field show that the various buildings, stories and local wisdom about disasters held by the people of Central Sulawesi can be important tools for building awareness, increasing preparedness, managing trauma and strengthening social solidarity. Maurice Halbwachs also explained that memory is never purely individual. It is always formed in a social context, through interactions with groups and social frameworks that give meaning to past experiences (Halbwachs 1980, 1992). In the context of the disaster in Central Sulawesi, Halbwachs’s concept is relevant: how communities remember disasters, what they choose to remember and how they pass those memories on to the next generation are all shaped by specific socio-cultural frameworks.

According to a local scholar, there are three important elements in the collective memory process: continuity of memory, awareness and the political will of the people to preserve their memories (Wattimena 2016). Firstly, collective memory is dynamic. It is an ongoing process. It can change in line with changes occurring in society.

Secondly, a society’s collective memory is embedded in the consciousness of its citizens. Thirdly, the preservation of collective memory is highly dependent on the political will of its citizens. This means that collective memory is rooted in the collective consciousness of a society’s citizens. It also becomes part of that society’s collective identity (Wattimena 2016). From this explanation, it can be understood that in the context of the disaster in Central Sulawesi, the fading of the community’s collective memory is caused by a lack of these three elements: continuity of memory, awareness and the political will of the local community to preserve their memories. The people of Central Sulawesi, rich in traditional cultures related to disasters, were still unable to utilise such memory in facing the earthquake, tsunami and liquefaction disaster that occurred on 28 September 2018.

Finally, one of these narratives and Halbwachs’s most important insights is that collective memory is always oriented towards the present and the future. Humans sometimes do not remember the past for its own sake, but because of its relevance to the group’s present needs, identity and goals. Therefore, the disasters experienced by our predecessors should serve as reminders for local communities and as capital for community resilience, enabling them to coexist with natural disasters.

## Conclusion

Collective memory of the disaster in Indonesia, particularly the province of Central Sulawesi, is a complex and multi-layered phenomenon. It lives on in place names, ancestral stories, traditional rituals and even in the concrete traditional cultures among the people of the province. Both forms of memory (traditional cultures and buildings) have their own strengths and limitations. However, the traditional cultures regarding disasters owned by the Kaili community only started to be widely discussed after the 2018 disaster. Before the disaster, local wisdom was not a concern of the community. The lack of public interest in traditional cultures, including local languages, is the reason why local wisdom is not popular among local people. Moreover, the oral tradition of Kaili, without the support of written culture, is another obstacle to preserving local wisdom. The 2018 disaster was a tragic reminder of what happens when collective memory erodes. Fortunately, the information digitisation system through the Internet has been helpful currently in answering the limitation of the cultural preservation. Several *kayori* texts and folklore about disasters have been documented in text and video form by several parties via various platforms on the Internet. Even though the number is still very limited, it can reduce pessimism about the fading of Central Sulawesi’s traditional cultures. Eventually, the disaster in Central Sulawesi and Maurice Halbwachs’ idea of collective memory teach us that memory is a social project that is constantly being constructed and reconstructed. It is never final, never complete and always open to interpretation and reinterpretation. In the context of disasters, collective memory serves as a bridge between a traumatic past and a safer future, provided we are willing to learn from it. The challenge for Central Sulawesi and for all of disaster-prone Indonesia and the world is how to preserve and pass on this collective memory in an authentic, inclusive and effective way. Ultimately, the memory of disasters will not paralyse with trauma but empower with knowledge and preparedness.
